# Posture in Acute Gastric Dilatation

**Published:** 1915-05

**Authors:** 


**Posture in Acute Gastric Dilatation.** Linke, Beitráge zur Klin. Chir. Vol. 93, No. 2. Having lost two cases, the third patient was placed face downward, experienced almost immediate relief and recovered. (Note. Do not forget the tendency to term acute gastric dilatation, what is merely distension, due to obvious causes, especially when a pre-existing moderate gastric dilatation has gone undiscovered. The method is good, however, and even applicable in non-surgical cases. Ed.)

